# Impact of the COVID-19 Pandemic on the Education of Plastic Surgery Trainees in the United States

**DOI:** 10.2196/22045

**Published:** 2020-11-17

**Authors:** Alireza Hamidian Jahromi, Alisa Arnautovic, Petros Konofaos

**Affiliations:** 1 Department of Plastic Surgery University of Tennessee Health Science Center Memphis, TN United States; 2 Department of Plastic Surgery Rush University Medical Center Chicago, IL United States; 3 The George Washington University School of Medicine and Health Sciences Washington, DC United States

**Keywords:** COVID-19, coronavirus, education, plastic surgery residency, plastic surgery fellowship, surgery residency, impact, trainee

## Abstract

The current COVID-19 pandemic has vastly impacted the health care system in the United States, and it is continuing to dictate its unprecedented influence on the education systems, especially the residency and fellowship training programs. The impact of COVID-19 on these training programs has not been uniform across the board, with plastic surgery residency and fellowship programs among the hardest hit specialties. Implementation of social distancing regulations has affected departmental educational activities, including preoperative, morbidity and mortality conferences and journal clubs; operating room educational activities; as well as the overall education of plastic surgery trainees in the United States. Almost all elective and semielective surgeries across the United States were suspended for a few months during the COVID-19 pandemic; this constitutes a significant portion of plastic surgery cases. Considering the current staged reopening policies, it may be a long time, if ever, before restrictions are completely lifted. In this paper, we review the multidimensional impact of the current COVID-19 pandemic on the training programs of plastic surgery residents and fellows in the United States and worldwide, along with some potential solutions on how to address existing challenges.

Education is the passport to the future, for tomorrow belongs to those who prepare for it today.

Malcolm X

The current COVID-19 pandemic has vastly impacted health care in the United States and globally, and it is continuing to dictate its unprecedented influence on education systems, especially the health system and medical education field (ie, residency training programs). The impact of COVID-19 on residency and fellowship trainings has not been uniform across the board. Among the various programs, plastic surgery residency and fellowship programs are one of the hardest hit specialties. Almost all elective and semielective surgeries across the United States were suspended for a few months due to COVID-19. Considering the current staged reopening policies, it may take a long time, if ever, for restrictions to be completely lifted. Suspensions of various activities have considerably affected teaching opportunities for plastic surgery residents during this time, as many plastic surgery cases are elective and semielective in nature. Learning operative skills is a core aspect of education in the surgical specialties, which makes these surgical specialties more vulnerable than their medical counterparts in the COVID-19 era. In many instances, surgical residents have been left out of the operating room (OR) altogether, whereas in some others, they have instead been assigned to screening facilities, critical care facilities, and emergency rooms, wherein currently there is the greatest need for physicians and residents to staff undermanned hospitals and clinics [1]. Although surgical residents may find this somewhat problematic at the time, these assignments are absolutely necessary and can help them enhance their experiences and training in any future health crises. Moreover, these experiences will allow them to diversify their level of expertise and confidence in working in similar environments during times of uncertainty or health catastrophes in the future.

Overall, the impact of the limitation in operative or surgical exposure for residents due to COVID-19 is dependent on the level of the trainee (greatest for senior and impending graduate residents and fellows) and the general length of the training program (greatest for those enrolled into shorter training programs, ie, 1-year fellowships) [1]. For example, for a trainee in a 1-year fellowship (gender affirmation surgery, aesthetic surgery, or reconstructive microvascular surgery), the training opportunities lost and clinical activities curtailed between March and June 2020 due to the COVID-19 pandemic would account for about one-third of their entire fellowship training period, which would be difficult to compensate for [1].

Furthermore, because of social distancing guidelines, many in-person opportunities that enable plastic surgery residents to interact with attendings—a vital component of residency education—have been discontinued. The effects of these guidelines have been very drastic and may even be augmented if more waves of COVID-19 with subsequent restrictions and surgery suspensions arrive in the future. It is, therefore, necessary and important to analyze the educational impact of COVID-19 on plastic surgery residency and fellowship and to propose ways to address this challenge.

Certification in plastic surgery is possible through 2 avenues: an integrated pathway of plastic surgery residency (which is a 6-year training program) and an independent pathway that includes satisfactory completion of a formal training and board eligibility in general surgery, otolaryngology (ENT), neurosurgery, orthopedic surgery, urology, or oral maxillofacial surgery residency (which ranges from 5 to 7 years) followed by a 3-year plastic surgery fellowship. Oral maxillofacial surgery graduate candidates must complete 2 extra years of general surgery training in addition to an MD or DDS. For program accreditation based on Accreditation Council for Graduate Medical Education (ACGME) requirements, the annual review process of the residents and fellows and their minimum case logs requirements must all be met. However, many residents and fellows will likely not be able to complete the required number of operative assignments, clinical rotations, and patient care encounters due to COVID-19. Hence, the ACGME has given program directors the right to assess the competence of residents and fellows during COVID-19 to determine whether that specific individual has met the minimum competency to graduate and practice their specialty unsupervised [1,2]. Although the current circumstances due to COVID-19 necessitate program directors to make such assessments in light of reduced semielective and elective cases, this process recognizably has a few issues. First, certain parts of the United States have been disproportionately affected by COVID-19. Some residents and fellows may still be assigned to screening facilities, critical care facilities, and emergency rooms, whereas other trainees may currently be able to perform semielective and elective procedures, depending on local and state laws. Residents in both these scenarios would have had varying surgical exposure; as a result, the assessment of their competence in surgery or plastic surgery will have considerable differences, to no fault of their own. Second, this change also adds in a subjective component to how trainees are assessed, rather than relying on an objective requirement of minimum case logs to designate competence. Subjective evaluations always lead to differences in interpretation. At present, the real impact of this decision on surgery residents and fellows remains unknown, and whether this change in decision-making will have an impact on their eventual practice until the distant future cannot be ascertained.

In the United States, plastic surgery residency and fellowship training comprises 3 types of educational activities for trainees to achieve certification and graduate-level knowledge and skills: (1) didactic activities, including textbook and journal reading assignments; (2) departmental educational activities, including preoperative and morbidity and mortality conferences and journal clubs; and (3) OR educational activities. Each of these categories of educational activities has been affected by COVID-19 through multiple facets. The aforementioned activities are set to equip the candidates with the 6 core competencies set by the ACGME: (1) practice-based learning and improvement, (2) patient care and procedural skills, (3) systems-based practice, (4) medical knowledge, (5) interpersonal and communication skills, and (6) professionalism. Although solutions for each type of educational activity have been proposed currently, more insight into this escalating issue will be needed in the future. Through the didactic educational method or activity, program directors and attendings distribute reading and teaching materials (such as journal articles, operative case or technique video clips, posters, and textbook chapters) to trainees, allowing them to learn individually, in an unstructured manner. Then, residents or fellows and attendings convene in-person to discuss these materials in one-on-one or group discussion sessions. Due to social distancing, many of these in-person meetings have been cancelled, negatively affecting didactic learning opportunities for plastic surgery residents and fellows. However, this issue can be easily addressed, for the most part, with online technologies, such as Zoom, Skype, or WebEx. Through these virtual meetings, trainees and faculty physicians can gather via an online platform to discuss didactic materials. Not only does this practice abide by social distancing guidelines, but it allows for greater flexibility in scheduling and avoids travel cost, on-site hazards, and wasted time, thereby increasing convenience for everyone involved as they no longer have to convene in a common room. Thus, in the post–COVID-19 era, this may become the preferred method for didactic activities for residents and fellows.

Nevertheless, as many programs have not previously utilized remote conferencing for didactic educational methods, the quality of instruction and education will likely be affected during the learning curve of adapting to virtual meetings [1]. This would also require a number of essential preparatory steps such as local connection logistics; software download; system compatibility assessment with individuals’ personal computers, laptops, and smart phone terminals; and security and privacy measures to avoid potential HIPAA (Health Insurance Portability and Accountability Act) violation. The aforementioned virtual platforms (Zoom, Skype, and WebEx) also pose potential security risks, especially with HIPAA-sensitive information. Participants should be required to enter a meeting password when joining, which is provided by the host beforehand in order to prevent hackers from joining the call or spreading malware and viruses to participants’ devices. Additionally, Zoom has also made end-to-end encryption available for all users, but this feature disables the participants from dialing-in via phone (they must use a computer), thus compromising user convenience [3]. Some virtual platforms (WebEx and Skype) also allow recording of the sessions, which could be problematic if the recordings are retrieved or accessed by a third party. Although there are obvious security concerns associated with using these virtual platforms, users have no alternative to using them to comply with social distancing guidelines. Nevertheless, secure conferencing must be of utmost importance, especially when sensitive information is being shared with regard to patient information.

From an educational standpoint, if participants turn off their video and microphone during these virtual calls, this allows for complete withdrawal from virtual learning [4]. However, in a neurology residency program, Morawo, Sun, and Lowden [4] were able to successfully stimulate viewer engagement in a virtual learning environment by utilizing retrieval practice questions asked via the interactive feature Poll Everywhere and a group quiz competition. Additionally, Zingaretti et al [5] reported that although many surgery residents have been using webinars during the pandemic, online technologies are beneficial but not sufficient given the complexity of plastic surgery topics. Nevertheless, using online technologies for virtual learning provides residents and fellows an opportunity to share information with other trainees worldwide. For example, online educational conferences or lectures by attendings could be opened up to participants in other countries or programs. Rare cases or surgeries could also be shared online to educate other trainees that may not have access to these cases in other areas of the world. Overall, live discussions and interactions, including point or counterpoint arguments, cannot be fully replaced with online technologies especially in complex fields like plastic surgery, but these online alternatives can supplement training in the current times.

The departmental educational activity component of the resident program consists of in-person faculty lectures, journal clubs, grand rounds, morbidity and mortality, and preoperative conferences. The same aforementioned didactic methods can be employed for department-specific residency and fellowship meetings. Other approaches to educational activities can also be utilized, such as texting-based educational material, but the costs and benefits of this technique must be considered. Clavier et al [6] investigated the distribution of educational documents via WhatsApp, an instant messaging app, instead of traditional online learning platforms for anesthesia residency programs. Younger generations are likely familiar with WhatsApp and open to using it as an educational platform. However, a previous study found that residents in the traditional learning group demonstrated higher medical reasoning than those in the WhatsApp learning group, although no differences in medical knowledge were observed between the two groups [6].

Similarly, Savoy et al [7] investigated the use of texting-based educational material in a general surgery residency program. Texts were sent to medical students about surgery rotations and to general surgery residents about observed cases or patients during rounds. Although this study was conducted at a single institution, the results suggested that students from both study groups favored text messaging for educational purposes [7]. This form of education serves as “academic epinephrine” because an educational stimulus is prompted when the student is not anticipating it [7], indicating this could be a valuable tool for departmental educational activities. Nevertheless, using mobile phones for educational purposes may lead to distractions during dedicated educational periods, clinical duties, or operative time, which should also be taken into consideration.

The third component of plastic surgery residency and fellowship is OR education, which includes preoperative evaluation of patients (marking and planning for surgery), postoperative rounds, performing or assisting in the operative procedure, live examination of patients and their wounds, and in-person review of radiological and laboratory studies with respective subspecialists. Plastic surgery operative cases can be divided into 3 categories: emergency, semielective, and elective, all of which require a minimum case log for all residents and fellows. Emergency cases, across all operative specialties, have and will continue to occur regardless of the pandemic, as emergency cases have not been impacted or are less impacted by COVID-19. However, emergency cases may be reduced or more staggered owing to limited hospital bed capacity during COVID-19 times, which is likely dependent on the hospital location and the local number of COVID-19 cases. Moreover, with quarantine regulations and many people being furloughed, losing their jobs, or working from home, some may argue that overall emergency or trauma-related plastic surgery operations have reduced during the last few months, since around the start of the COVID-19 pandemic. In some institutions, rotating shifts have been established for residents based on the current, and likely reduced, surgical schedule to minimize the risk of virus contraction [1]. As long as trainees have received proper training and adequate personal protective equipment (PPE), it should be safe for them to continue to partake in emergent plastic surgery cases. During the exponential phase of COVID-19, some hospitals imposed restrictions on the number of surgical apprentices who could scrub on a case. In the future, it may be beneficial to video-record emergent cases to play for other residents who are unable to scrub in during emergent cases, so that their training is not completely compromised.

The second category of plastic surgery cases is semielective, which means the surgery is not emergent but must be performed to save a patient’s life eventually. In many hospitals, semielective surgeries are not being performed since a period of time based on recommendations of the American College of Surgeons *COVID-19: Elective Case Triage Guidelines for Surgical Care* [8] and the American Society of Plastic Surgeons [9] to cease elective and nonessential surgeries. Nevertheless, given the wide range of conditions that can be managed by plastic surgery, it is imperative, now more than ever, that residents and fellows continue to nurture their surgical skills during COVID-19 despite lack of opportunities to partake in semielective and elective surgeries. Potential methods to do so are to increase relevant reading material for plastic surgery residents; watch surgical videos; and train on mannequins, laboratory live animals, cadaveric animal parts, or training platforms. Such initiatives could help ensure that the residents’ skills are not negatively impacted by the COVID-19 pandemic [10].

Finally, elective surgical cases are a vital component of plastic surgery residency and fellowship programs. In certain areas of the United States, semielective procedures may be taking place or may have been reintroduced, but elective surgeries were cancelled indefinitely all over the country. To preserve residents’ surgical skills, the methods aforementioned for semielective procedures can be employed for elective surgeries (ie, continuing relevant readings, watching surgical videos, and using mannequins or cadaveric animal parts for surgical training). However, training in elective procedures will continue to remain a challenge for an unknown period of time, specifically in cities that are disproportionately affected by COVID-19, such as New York City, Boston, New Orleans, Philadelphia, Michigan, Chicago, and Washington D.C. Additionally, rhinoplasty, nasal reconstructive surgery, and other head and neck aesthetic surgeries (eg, brow lift and blepharoplasties) are very common elective procedures for plastic surgeons, and it is important to consider the potential aerosolization of virus particles during these operations [11]. Thus, if COVID-19 continues to remain prevalent, special precautions must be taken to minimize transmission of the virus; these include limiting the number of OR personnel, utilizing proper PPE and powered air-purifying respirators, and requiring patients who are undergoing surgery to report at least two or three consecutive COVID-19 negative tests in order to decrease the possibility of false-negative results [11].

With the special precautions necessary for specific preoperative evaluations of patients undergoing elective surgeries during this time, a number of challenges arise with the reintroduction of elective surgeries and the protocols required for them. Prior to surgery, patients must have at least one and preferentially two negative COVID-19 tests, with the most recent negative test taken 24-48 hours prior to surgery; however, this criteria requires patients to come in for their surgery early to be tested for COVID-19 [12]. Unfortunately, having to undergo 2 tests translates to an increase in travel time and a possible loss in work time, but it is necessary as the reverse transcription polymerase chain reaction (RT-PCR) test for COVID-19 is known to have up to 30% false-negative rate [13]. The RT-PCR test requires a nasopharyngeal or oropharyngeal swab sample, which are uncomfortable for the patients. It may also be difficult to actually acquire a viable sample, thereby contributing to the high false-negative rate. Furthermore, some testing centers may not test asymptomatic walk-in patients, which would require a preoperative testing request and coordination from the referring physician as well. Patients who have at least two negative COVID-19 tests should self-quarantine in their homes 24-48 hours prior to surgery, isolating from the family who could be potential carriers of the virus–doing so may be difficult for the patient and challenging for plastic surgeons to enforce [12].

Physicians, trainees, nurses, and hospital employees should also be regularly tested because they could be asymptomatic carriers, too. As elective procedures constitute a large part of plastic surgery (and physician’s incomes), health care professionals may be less likely to report their symptoms or viral status in fear of having to cancel these elective procedures. Even with the testing protocol in place, there is still room for unpredictability and risk for viral transmission due to the high false-negative rate [12]. Although the false-positivity of COVID-19 tests is not well discussed, a false-positive test could cause financial burden for the patient and the health care system, and it may add to the complexity of the situation.

Surgical facilities must also have designated employees who screen patients on the phone beforehand and assess patients’ potential risk of carrying the virus or having COVID-19 (by asking questions about symptoms, recent travel, close contacts, body temperature, respiratory symptoms, etc), which takes up time that the employee would have otherwise spent on performing other tasks [12]. Other factors to consider are the cost of proper PPE for all OR employees and hospital staff and provision of a functional powered air-purifying respirator device for each OR (which would imply additional costs if the hospital does not already own them, an issue for hospitals already strained on resources during this time). Patient surgical risk stratification is also important when deciding whether an elective procedure should be performed. Patient age, comorbidities (diabetes, hypertension, obesity, lung diseases, etc), and type or complexity of surgery must be considered [12]. Finally, even after the COVID-19 pandemic eases out, the virus will continue to exist, and although elective procedures form a substantive part of plastic surgery training and should eventually be reintroduced, it is vital that these prevailing challenges be acknowledged and addressed going forward**.**

The lack of elective surgical cases for residents and fellows may be further exacerbated in the future if there is a second wave resurgence of COVID-19 in the fall during the flu season, or further ahead into 2021 and beyond. Thus, the COVID-19 pandemic undoubtedly has had a negative impact on plastic surgery training and its effects in America remain ever-evolving. The European Academy of Facial Plastic Surgery Task Force [11] currently suggests using technology, such as surgical videos and webinars, as a teaching tool for residents and fellows during this time in order to protect residents from contracting the virus, conserve the limited PPE, and maintain their surgical skills. Other virtual tools that could be used for plastic surgery education include the Anatomage Table and Touch Surgery. The Anatomage Table allows for an advanced, 3D virtual dissection of a life-sized human cadaver, contributing to a more precise visual perception of the human body, which may not be available otherwise during COVID-19 [5]. Touch Surgery is a surgical application software that comprises 42 plastic surgery procedures for residents or fellows to watch [5]. These electronic tools cannot replace firsthand surgical experience; however, they can aid in surgical preparation and confidence for trainees during the pandemic.

Although the case log minima for residency and fellowship graduates during this time period will likely be interpreted in the context of the effects of COVID-19 on that specific program [1,2], more suggestions, creative ideas, and solutions are necessary to address the lack of surgical training in elective cases for plastic surgery residents. This pandemic will also lead to a backlog of facial and elective plastic surgery cases [10], which is another important consideration that will have to be addressed in the post-COVID-19 era. In other words, patients and their plastic surgery pathologies will wait to be addressed at the appropriate time, but they will by no means disappear.

Currently, there has been little research on how COVID-19 has affected medical residencies, and even less information pertaining to plastic surgery residencies and fellowships is available pertaining. The time a resident or fellow takes to mature, with their training culminating into the completion of their education, producing a capable, confident, and well-trained specialist has been compromised by the ongoing pandemic. The safety of patients, residents, fellows, and attendings is of utmost importance; nevertheless, the educational impact of COVID-19 on plastic surgery residency and fellowship is potentially devastating for future generations of the specialty and cannot be ignored. Additionally, the future of COVID-19 and its duration are unknown, which is why it may have a lasting impact on health care provision in the United States for many months and years to come. Therefore, more alternatives must be explored and utilized in plastic residency and fellowship programs in terms of didactic activities, departmental educational activities, and OR educational activities so residents and fellows receive adequate training and are confident in their surgical skills upon graduation.

## Abbreviations

ACGME: Accreditation Council for Graduate Medical Education
HIPAA: Health Insurance Portability and Accountability Act
OR: operating room
PPE: personal protective equipment
RT-PCR: reverse transcription polymerase chain reaction
